# Sub-ppb Level Detection of BTEX Gaseous Mixtures with a Compact Prototype GC Equipped with a Preconcentration Unit

**DOI:** 10.3390/mi10030187

**Published:** 2019-03-13

**Authors:** Irene Lara-lbeas, Alberto Rodríguez-Cuevas, Christina Andrikopoulou, Vincent Person, Lucien Baldas, Stéphane Colin, Stéphane Le Calvé

**Affiliations:** 1 ICPEES UMR 7515, Université de Strasbourg/CNRS, F-67000 Strasbourg, France; ilaraibeas@unistra.fr (I.L.-l.); candrikopoulou@unistra.fr (C.A.); 2 Institut Clément Ader (ICA), Université de Toulouse/CNRS, INSA, ISAE-SUPAERO, Mines-Albi, UPS, 31400 Toulouse, France; baldas@insa-toulouse.fr (L.B.); stephane.colin@insa-toulouse.fr (S.C.); 3 In’Air Solutions, 25 rue Becquerel, 67087 Strasbourg, France; arodriguez@inairsolutions.com (A.R.-C.); vperson@inairsolutions.fr (V.P.)

**Keywords:** preconcentrator, microfluidics, miniaturized gas chromatograph, BTEX, PID detector

## Abstract

In this work, a compact gas chromatograph prototype for near real-time benzene, toluene, ethylbenzene and xylenes (BTEX) detection at sub-ppb levels has been developed. The system is composed of an aluminium preconcentrator (PC) filled with Basolite C300, a 20 m long Rxi-624 capillary column and a photoionization detector. The performance of the device has been evaluated in terms of adsorption capacity, linearity and sensitivity. Initially, PC breakthrough time for an equimolar 1 ppm BTEX mixture has been determined showing a remarkable capacity of the adsorbent to quantitatively trap BTEX even at high concentrations. Then, a highly linear relationship between sample volume and peak area has been obtained for all compounds by injecting 100-ppb samples with volumes ranging from 5–80 mL. Linear plots were also observed when calibration was conducted in the range 0–100 ppb using a 20 mL sampling volume implying a total analysis time of 19 min. Corresponding detection limits of 0.20, 0.26, 0.49, 0.80 and 1.70 ppb have been determined for benzene, toluene, ethylbenzene, m/p-xylenes and o-xylene, respectively. These experimental results highlight the potential applications of our device to monitor indoor or outdoor air quality.

## 1. Introduction

In recent years, there has been an increasing interest in air pollution since numerous studies have demonstrated its impact on human health [1,2,3]. Among the broad variety of identified air contaminants, benzene, toluene, ethylbenzene and xylenes (BTEX) require particular attention not only for their toxic, mutagenic, and/or carcinogenic effects but also for their key role in photochemical reactions. These compounds can be emitted by several indoor as well as outdoor sources. In outdoor air, the main sources are related to fuel storage or combustion processes such as road traffic, petrol stations or industrial activities [4,5,6,7]. In closed environments, most of BTEX are emitted by cleaning products, furnishings as well as building materials like flooring materials, wall coverings, adhesives, varnishes or paints [8,9,10]. Smoking and cooking are also considered as other major sources of BTEX in indoor air [11,12]. Numerous investigations have demonstrated the harmful effects of BTEX on human health even at low concentrations [13]. Fatigue, loss of coordination, memory problems, headache, skin and eyes irritation as well as other more severe effects like asthma, kidney damage or neurological problems have been linked to BTEX exposure [14,15]. In addition, benzene is a known carcinogen compound, with long-term exposure being associated with the development of leukaemia [16]. Thus, in 2013, a threshold limit value of 5 µg m^−3^ (1.6 ppb) for benzene was set by the European Union in public buildings. In France, this limit was decreased to 2 μg m^−3^ (0.6 ppb) in 2016. Therefore, to check if indoor air quality (IAQ) is in accordance with the new legislation, on-site rapid and sensitive analysis is required.

So far, methods based on gas chromatography have typically been the techniques employed for BTEX analysis in indoor and outdoor air. Benchtop chromatographs are sensitive and accurate instruments enabling BTEX detection in the order of parts per trillion (ppt). However, their large size, heavy weight and high energy consumption limit their use for in-situ measurements. Consequently, many air quality measurement campaigns have been conducted using commercial sampling cartridges [8,17,18,19,20,21] that were subsequently analyzed in a laboratory increasing not only the total analysis time but also the risk of sample degradation during storage or transport. Furthermore, these off-line analyses do not provide concentration-time profiles since each measurement represents an average value of pollutant concentration over the selected sampling time.

During the past two decades, great efforts have been made to develop real-time sensitive miniaturized gas chromatographs. Portable instruments containing micro-electro-mechanical systems (MEMS)-based components have grown in popularity due to their small dimensions, low energy consumption and short time of analysis. Table 1 summarizes the most remarkable commercially available and laboratory prototypes of miniaturized gas chromatographs (GC) developed in the last decade. All devices presented are highly portable (weight < 5 kg) enabling on-site measurements. Most of these instruments perform BTEX analysis in less than 15 min providing high-resolution concentration-time profiles. Nevertheless, these analyzers have a detection limit of a few ppb, which is not enough to comply with the requirements of the new regulation. So far, only the GC-metal oxide semiconductor (MOS) reported by Zampolli et al. [22] and the GC- photoionization detector (PID) developed by Skog et al. [23] were able to detect concentrations below 1 ppb. High sensitivities are usually correlated with long sampling times. In the first case, 55 min of sampling and around 12 min for separation were needed to achieve sub-ppb detection of BTEX [22]. Shorter sampling (20 min) and similar separation (15 min) times were required by Skog et al. [23] to reach limits of detection (LOD) in the order of ppt. Both devices imply a considerable long total analysis time, hindering the possibility to provide near real-time BTEX concentrations. Regarding the MEMS-based instruments available in the literature, a sensitive and rapid BTEX analysis remains a challenging issue.

The present work reports the development of a compact GC prototype for near real-time BTEX detection at sub-ppb levels and its validation under controlled laboratory conditions. The device is based on the miniaturized GC developed by Nasreddine et al. [24] where a preconcentration unit has been added in order to significantly improve its sensitivity. An improvement ratio between 1.55 and 4.46 in detection limit was achieved for the different compounds compared to the previous version. To our knowledge, this is the first work to report a GC prototype able to perform sub-ppb BTEX levels detection in such a short analysis time.

The paper is organized as follows: Section 2 describes the GC working principle and presents the experimental setup for BTEX generation employed in this project. Section 3 presents the performances of the GC prototype in reference to adsorption capacity, linearity and sensitivity. Section 4 summarizes the conclusions and proposes several improvements for the future work.

## 2. Materials and Methods

### 2.1. Prototype of Micro Gas Chromatograph (GC)

**Figure 1:**
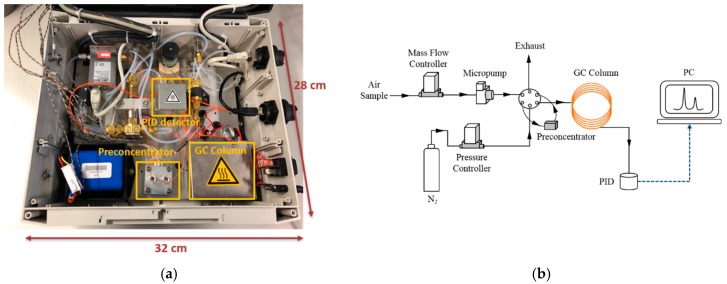
(**a**) Photograph of the compact gas chromatographs (GC) prototype and (**b**) schematic view of the device updated with a preconcentration unit.

The GC laboratory prototype employed in this study is presented in Figure 1. The system is based on the GC laboratory prototype developed by Nasreddine et al. [24]. In the present work, a preconcentration module has been integrated into the system enabling an improvement in sensitivity. The detailed operating principle of the GC is described in [24] so that only a brief description of the overall system is given below while the preconcentration module developed in the present work is highly detailed in the next section. The system operates according to four steps: sampling, preconcentration, separation and detection. Samples are collected and introduced in the preconcentrator (PC) by means of a SP 570 EC-BL micropump (Schwarzer Precision, Germany) and a EL-FLOW flow controller (Bronkhorst, Ruurlo, Netherlands). Separation step is carried out using a commercial 20-m long capillary column (i.d. 0.18 mm, RXi-624 stationary phase, 1 µm film thickness, Restek, Bellefonte, PA, USA). Polydimethylsiloxane (PDMS) columns are typically used for BTEX separation because as they are non-polar, they provide high resolution but long separation times as well. In environmental monitoring, a compromise between short analysis time and reasonable resolution is required, therefore, a slightly more polar column (Rxi-624Sil MS) was selected. Detection is conducted employing an eVx Blue mini photoionization detector (PID) (Baseline MOCON, Lyons, CO, USA) equipped with a 10.6 eV ultra-violet lamp. This GC laboratory prototype was controlled by a computer using a homemade software.

In a previous study [24], injection time was set to 20 s to transfer the sampling loop content to the column. In this new version, injection time of 80 s has been fixed according to the temperature ramp of the heating system, where 150 °C were reached in 60 s and were maintained for 20 s. After desorption, the six port-valve returned to the initial position to start the next sampling.

In the aforementioned work [24], flow rate of 2.5 mL min^−1^ and 80 °C were selected as the optimal conditions for BTEX separation. Using these conditions, BTEX analysis was performed in 10 min and detection limits between 1–3 ppb were found for the different compounds. Analytical performances of this device were validated under controlled laboratory conditions and in real environments [36,37]. In the present version, as the enriched peaks are larger, temperature of the column had to be reduced of 10 °C to avoid coelution between ethylbenzene and m/p-xylenes peaks; therefore, the total analysis time was increased to 15 min.

### 2.2. Preconcentration Module

**Figure 2:**
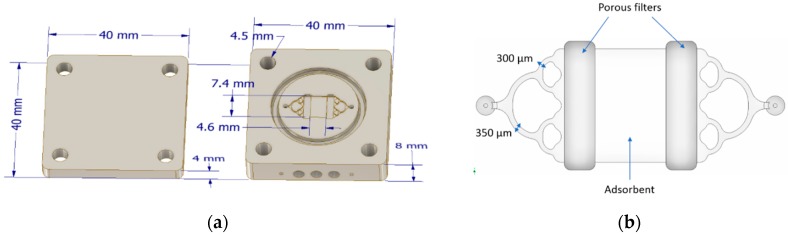
(**a**) Design of the preconcentrator and (**b**) zoom on the microfluidic system and the adsorbent cavity.

An aluminium PC with dimensions of 40 mm × 40 mm × 12.3 mm and weight of 54.9 g was manufactured and integrated into the GC laboratory prototype. The device is presented in Figure 2. The design is based on the preconcentrator proposed by Camara et al. [38] in which a symmetrical manifold fluidic system was fabricated at the inlet and outlet of the adsorbent cavity to promote a uniform flow distribution. However, there are some differences between both designs. In this PC, two metal porous filters (GKN Sinter Metals, Bonn, Germany) are located between the channel system and the microfluidic cavity to ensure that the adsorbent remains in the cavity and to prevent clogging of the microchannels. The manifold consists of one inlet channel split in two channels of 350 µm which are also split to obtain finally four channels of 300 µm connected to a microfluidic cavity of 4.6 mm × 7.4 mm (see Figure 2b) where the adsorbent is placed. In this cavity, 5.8 mg of Basolite was packed manually. Heating system consists of three heating cartridges (Watlow, St. Louis, MO, USA) of 70 W each. The system allows to reach a temperature ramp of about 150 °C/min. In the experiments, desorption was performed at 150 °C and this temperature was maintained for 20 s. Temperature is measured with a type K thermocouple (RS Components SAS, Beauvais, France). Prior to the beginning of the experiments, the adsorbent was conditioned at 180°C under a nitrogen flow of 10 mL/min during 2 h.

### 2.3. Experimental Setup for BTEX Generation

**Figure 3:**
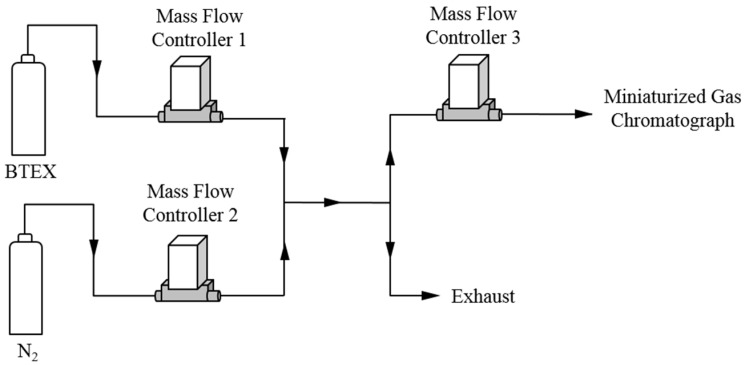
Experimental setup for BTEX generation.

Different BTEX concentrations were generated using the experimental device shown in Figure 3. A standard mixture of BTEX purchased from Messer (Folschviller, France) was diluted with nitrogen (99.999% purity) using mass flow controllers 1 and 2. The initial concentration of every compound was equal to 100 ppb with a 10% uncertainty. This setup allows generating different concentrations in the range 2–100 ppb. Mass flow controller 3 was used to select the sampling flow rate which was set to 5 mL/min for all the experiments.

### 2.4. Breakthrough Test

**Figure 4:**
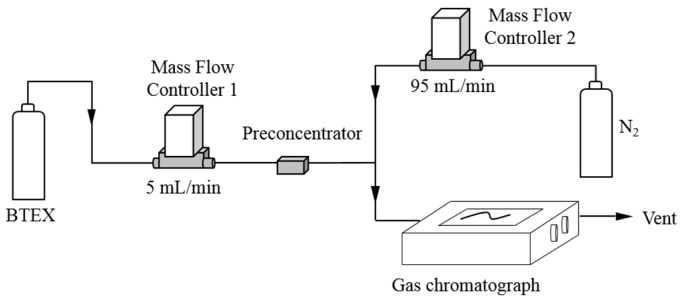
Schematic drawing of set-up used for breakthrough experiments.

The above described preconcentrator was mounted on the set-up showed in Figure 4. The 5 mL/min of an equimolar 1 ppm BTEX mixture diluted in nitrogen (Air Products SAS, Aubervilliers, France) flowed through the preconcentration unit. This concentration was too high to be analyzed by a standard gas chromatograph (µBTEX-1 In’Air Solutions, Strasbourg, France) equipped with a 200 µL sampling loop. Therefore, the gas stream from the preconcentrator outlet was diluted prior to the analysis using an additional flow of 95 mL/min of nitrogen (99.999% purity, Messer, Folschviller, France) to avoid saturation of the gas chromatograph detector. The whole diluted gas stream was connected directly to the chromatograph sampling loop which was constantly renewed. Every 11 min, the content of the sampling loop was injected into the separation column and analyzed.

## 3. Results and Discussion

Numerous experiments were conducted to evaluate the performances of the GC prototype: (1) A breakthrough experiment was carried out with a high BTEX concentration of 1 ppm in order to determine the adsorption capacity of the preconcentrator in the studied conditions; (2) repeatability was investigated; (3) the sample volume varied in the range 0–80 mL for a fixed gaseous concentration of BTEX; and (4) the gaseous BTEX concentration varied between 0–100 ppb for a given sample volume.

### 3.1. Adsorption Capacity

The adsorption capacity is dependent on the preconcentrator geometry, the adsorbent itself and the gas flow rate. The breakthrough time is defined as the time at which 5% of the molecules are leaving the adsorbent bed [39] so that it indicates the maximum sampling time to conduct a quantitative analysis at this concentration. Consequently, the breakthrough point is reached when 5% of the injected concentration (*C*_0_) is passing through the adsorbent and, thus, the measured concentration at the outlet (*C*) is equal to 0.05 *C*_0_. It should be noted that the breakthrough time will depend on the injected gas concentration, the flow rate and on the molecule in the case of a competitive adsorption.

Basolite C300 was selected as adsorbent as it has been already employed for benzene preconcentration in other analytical devices [40], demonstrating better preconcentration performance than Tenax TA, one of the most common materials for BTEX preconcentration.

**Figure 5:**
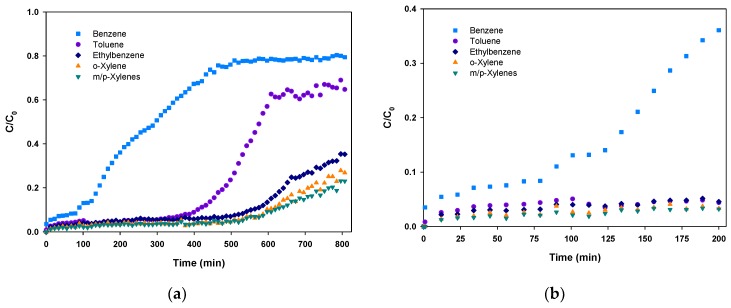
(**a**) *C/C*_0_ vs. time during a breakthrough experiment performed with an equimolar 1 ppm mixture of BTEX over Basolite-filled preconcentrator and (**b**) enlarged view of the first 200 min of the experiment.

In order to assess the adsorption capacity of the preconcentrator, a breakthrough experiment was performed. In this experiment, an equimolar 1 ppm (*C*_0_) mixture of BTEX flowed through the preconcentrator at a flow rate of 5 mL/min. Usually, BTEX concentrations in indoor air are lower than 10 ppb and do not exceed 100 ppb, so that 1 ppm BTEX mixture simulates a highly polluted environment. The effluent concentration (*C*) at the preconcentrator outlet was continuously analyzed by a BTEX analyzer provided by In’Air Solutions (µBTEX-1 In’Air Solutions, Strasbourg, France) and the relative BTEX concentrations (*C/C*_0_) were plotted versus time (see Figure 5a).

As it can be observed in Figure 5a, breakthrough curves have two different parts. The first part is almost a flat line indicating that the concentration at the outlet is close to zero and, thus, all the molecules are being adsorbed. Once the breakthrough time is reached (*C/C*_0_ = 0.05), the second step begins. In this interval, the adsorbent starts to be overloaded and some of the molecules are not being adsorbed, leading to an increase of concentration at the PC outlet. This concentration rise is sharp at the beginning and starts to be less pronounced as the preconcentrator is close to the total saturation. In our experiment, only benzene and toluene have reached this point with relative concentrations of 0.7–0.8. As the objective of this test was to determine the breakthrough time, the experiment was stopped before achieving the total saturation of the adsorbent (*C/C*_0_ = 1).

It is visible in Figure 5 that the adsorption capacity is remarkably different depending on the compound. Breakthrough times were determined as follows: 12 min (benzene), 156 min (toluene and ethylbenzene), 454 min (m/p-xylenes) and 457 min (o-xylene). The remarkable adsorption obtained for most of the compounds can be attributed to the large micropore volume of Basolite C300 as reported in other studies [41]. This noticeable adsorption capacity enables the collection of large sample volumes.

To illustrate the differences between benzene and the other compounds, Figure 5b presents an enlarged view of the first 200 min of the experiment where benzene clearly started to leave the adsorbent bed before the others. However, our results suggest that a sampling time of 12 min at 5 mL/min, corresponding to a sample volume of 60 mL, can be performed in a considerably polluted environment without any sample loss due to breakthrough, which would lead to an underestimation of the real gas concentration in air.

### 3.2. Repeatability

To evaluate the repeatability of the device, ten 20-mL samples containing 100 ppb of BTEX were consecutively injected. Between two consecutive injections, a cleaning step was conducted. During the separation step and once the sample was injected, the preconcentrator was maintained at 180 °C for 10 min to ensure there were no residues of the previous injection. After each cleaning step, a blank was conducted to verify that all BTEX were desorbed before starting the next injection. Peak area and retention time were determined for every replicate. Relative standard deviations (RSD) of peak area and retention time were calculated to verify the stability of the measurements. The corresponding RSD for the peak areas were 3.8, 6.3, 11.0, 14.7 and 13.4% for benzene, toluene, ethylbenzene, m/p-xylenes and o-xylene, respectively. The greater dispersion of ethylbenzene and m/p xylenes peak areas is probably due to the peak integration itself; as these species are coeluted, the error in the integration could be higher than in the case of benzene and toluene. Furthermore, in the current prototype, sample injection and desorption as well as the cleaning step were conducted manually because these functions were not yet integrated in the software although they can obviously impact the repeatability of the measurements. Indeed, it is expected to substantially improve the repeatability of the measurements when all the steps will be operated automatically by the software. Despite the lack of automatization, retention times were remarkably stable with RSD of 0.5, 0.7, 1.7, 1.4 and 1.4% for benzene, toluene, ethylbenzene, m/p-xylenes and o-xylene, respectively. Peak area of each blank was also determined, showing that after the cleaning step, the residues are less than 3% even after the injection of 100 ppb samples. It must be noted that a concentration of 100 ppb BTEX mimics a highly polluted environment where a preconcentrator is usually not required for analysis. Even if these results are far from ideal, the repeatability tests demonstrated that desorption is repeatable and, consequently, the aforementioned results show that the integration of the preconcentration unit into the previous GC version has not significantly influenced the repeatability of the device.

### 3.3. GC Signal Versus Sample Volume

**Figure 6:**
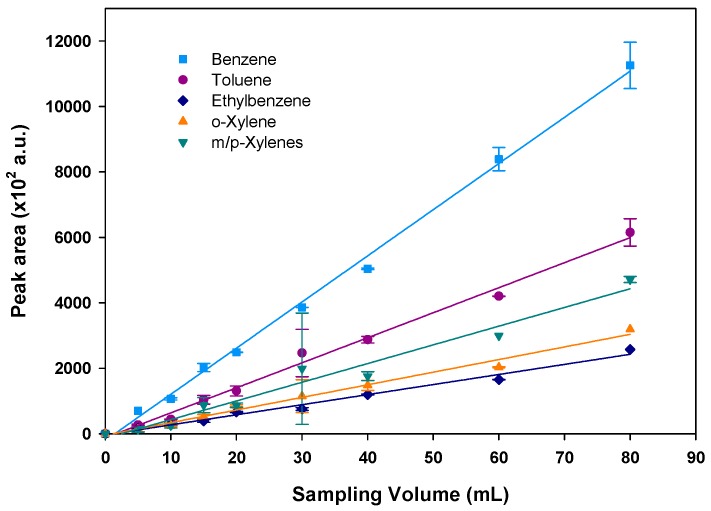
Peak area variation with sampling volume (BTEX concentration of 100 ppb). The vertical error bars show the standard deviation for duplicate injections.

After validation of the high adsorption capacity of BTEX on Basolite at high concentrations of 1 ppm and the stability of the measurements, the influence of sample volume passing through the preconcentrator was evaluated. For this, different volumes varying from 5–80 mL of a 100-ppb standard gaseous mixture of BTEX were injected in duplicate at a fixed flow rate of 5 mL/min. As it is described above, a cleaning step was performed after each analysis to ensure all BTEX have been desorbed. As illustrated in Figure 6, peak areas calculated from the chromatograms increase proportionally with the corresponding sampling volumes with correlation coefficients (R^2^) of 0.9946, 0.9892, 0.9824, 0.9608 and 0.9838 for benzene, toluene, ethylbenzene, m/p-xylenes and o-xylene, respectively. Therefore, different sample volumes can be used for the analysis in the studied range without modifying the performances of the experimental device. However, longer sampling improves the sensitivity but decreases the temporal resolution, i.e., the time needed for sampling and analysis increases. Therefore, a sample volume of 20 mL was selected as a compromise between sampling volume and time resolution, the flow rate being fixed at 5 mL/min to avoid any breakthrough.

### 3.4. Calibration Curves and Detection Limit

**Figure 7:**
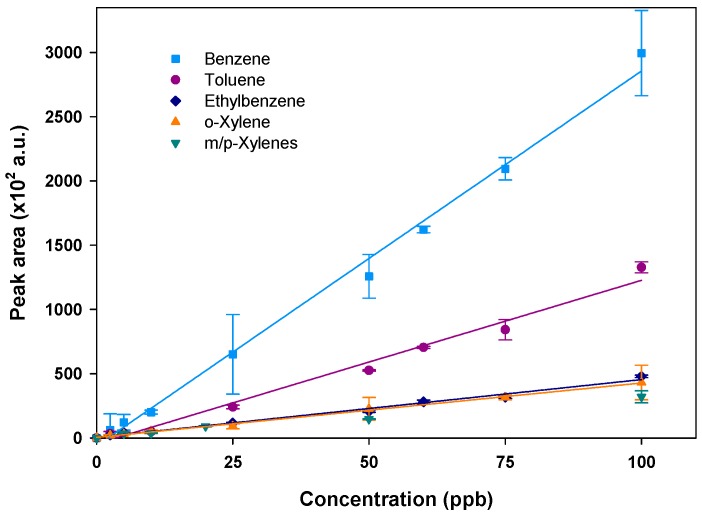
Calibration curves of BTEX performed at 5 mL/min and with a sampling volume of 20 mL. The vertical error bars show the standard deviation for duplicate injections.

Once adsorption capacity validated and sample volume fixed to 20 mL at a flow rate of 5 mL/min, a calibration was performed using different gaseous concentrations of the targeted compounds ranging from 2.5–100 ppb. Each concentration was injected in duplicate. As mentioned before, a cleaning step after each analysis was performed. The mean peak area was then calculated and plotted versus the injected concentration for each compound. Note that m- and p-xylenes were not separated. The obtained calibration curves are displayed in Figure 7 for each species.

The peak area increases linearly with the injected concentrations, demonstrating that the preconcentrator operates in a very satisfactory way as confirmed by the obtained correlation coefficients in the range 0.9777–0.9959 (see Table 2). The calibration slopes decrease with the molecular weight of the compound, being steeper for benzene (C_6_H_6_) and toluene (C_7_H_8_) than for the other compounds (C_8_H_10_) and, thus, leading to a greater sensitivity for the weakest compounds as already observed by Nasreddine et al. [24].

Detection limits were calculated from a signal-to-noise ratio of 3 for the two lowest injected concentrations (2.5 and 5 ppb). Using a sample volume of 20 mL, detection limits of the order of a few hundred ppt were obtained for all compounds, excepting o-xylene (see Table 2).

**Figure 8:**
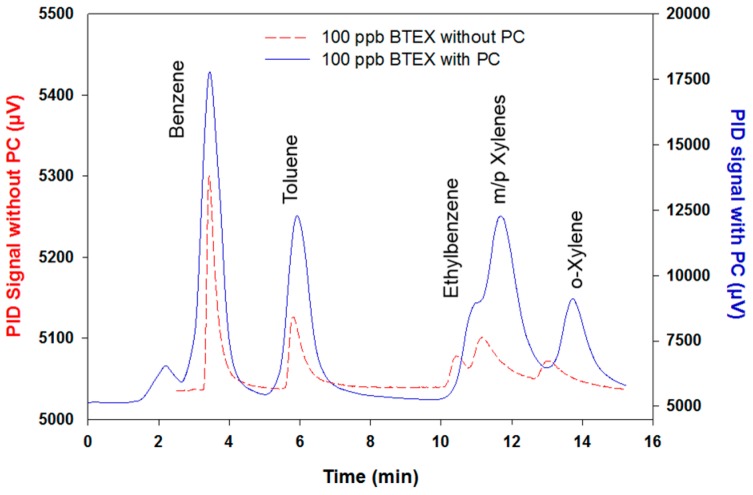
Red dashed line: Chromatogram of 100 ppb BTEX without preconcentration step (sample volume of 200 µL). Blue solid line: Chromatogram of 100 ppb BTEX with preconcentration step (sample volume of 20 mL).

These results show a considerable sensitivity enhancement compared to the previous development made by Nasreddine et al. [24] with an improvement ratio ranging between 1.55–4.46. To illustrate this improvement, a chromatogram of 100 ppb BTEX with and without the preconcentration stage is displayed in Figure 8. As evidenced by the figure, peak intensities are significantly greater in the new version, with the noise also increasing, but to a lesser extent.

It should be noted that lower detection limits can be achieved with this new prototype by increasing the sample volume passing through the preconcentration unit. In addition, the benzene limit of detection of 0.2 ppb obtained with the novel miniaturized GC integrating a preconcentration module is now consistent with the threshold limit value of 0.6 ppb imposed by the French regulation.

Apart from peak intensities, peak areas have been significantly enhanced. To quantify this improvement, a preconcentration factor (PF) has been defined as the ratio between peak areas with and without the preconcentration step. This factor is expected to be 100 as the sampling volume is increased by a factor of 100 (20 mL/200 µL) in the new prototype version. For the chromatogram presented in Figure 8, PF were 63.2, 82.4, 81.3, 89.3 and 77.9 for benzene, toluene, ethylbenzene, m/p xylenes and o-xylene, respectively. The difference between the obtained PF and the expected PF of 100 could be explained by two facts. Firstly, as it was demonstrated by the blanks performed in the repeatability test, some BTEX residues were not desorbed during the first desorption and, secondly, as the microfluidic cavity was manually packed with the adsorbent, it is probable that it was not completely filled and, thus, some BTEX molecules can pass through the cavity without being in contact with the adsorbent. For example, as illustrated in Figure 5b for the breakthrough experiments, some benzene molecules left the cavity without being adsorbed, even from the beginning of the experiments. This lower benzene trapping yield of 96% can explain the lower benzene PF of 63 observed. Nevertheless, satisfactory and repeatable results were obtained when varying the injected volume or concentration, showing that the trapping yield of BTEX and the preconcentrator factor are reproducible and have little significance in the accuracy of the analysis once the device is properly calibrated.

By sampling a volume of 20 mL, one full analysis cycle is performed in 19 min. Since preconcentrator cleaning and cooling down operated during the separation step, the resulting total analysis time was 19 min, which is acceptable to establish concentration–time profiles. Even if these results are successful, there are still significant scopes for improvements in terms of analysis time and peak resolution. It is expected to decrease the time of analysis by replacing the current separation column by a MEMS-based column enabling BTEX separation in 5 min, as it was reported in other studies [26,29]. Peak resolution can be enhanced by improving the performance of the PC heating system and/or reducing the detector response time. More compact devices with an integrated heating system can achieve a faster temperature ramp, which would result in shorter injection time and narrower desorption peaks. Additionally, a most powerful heating system could achieve higher temperatures in shorter times decreasing the amount of non-desorbed BTEX and, therefore, increasing the PF. Further work is planned to investigate alternative adsorbents that could potentially improve the trapping yield for benzene. On the other hand, a µPID detector with small detection chamber could also improve the peak resolution since the sample renewal inside the chamber would be faster and, thus, peak broadening would be reduced.

## 4. Conclusions

We reported the development of a compact GC prototype for near-real time BTEX analysis in sub-ppb range. Using a 20 mL sampling volume, BTEX analysis was conducted in 19 min and corresponding detection limits of 0.20, 0.26, 0.49, 0.80 and 1.70 ppb were calculated for benzene, toluene, ethylbenzene, m/p-xylenes and o-xylene, respectively. Considering the extremely low detection limit achieved by this prototype, it becomes possible to extend its use to other fields of application such as the food industry, early cancer diagnosis or explosives detection, by measuring other VOCs families and/or by changing the nature of adsorbent.

Several improvements can be proposed for future versions of the compact GC. First, the preconcentrator mass should be reduced in order to achieve faster heating, which would lead to more rapid temperature transfer to the adsorbent and, thus, faster desorption and then to thinner chromatographic peaks. Secondly, a shorter separation column based on MEMS technology could potentially reduce the total analysis time. In addition, the miniaturization of both components will reduce the energy consumption of the prototype, increasing its autonomy and improving its portability.

Finally, the automation of the device should integrate the cleaning step of adsorbent during pollutants chromatographic separation between two adsorption/desorption cycles, in order to improve the analysis quality.

## Figures and Tables

**Table 1:** Most representative compact gas chromatographs (GC) for BTEX analysis over last decade.

Ref.	Size (cm)	Weight (kg)	Sampling Time (min)	Analysis Time (min)	Preconcentrator *l* (mm) × *d* (µm) × *w* (mm)	Adsorbent	GC Column *l* (m) × *d* (µm) × *w* (µm)	Stationary Phase and Thickness	Carrier Gas Flow Rate (mL/min)	Detector	LOD (ppb)
This work	32 × 29 × 14	∼5	4	15	Cavity 4.6 × 350 × 7.4	Basolite C300 5.8 mg	Capillary 20 × 180 (i.d.)	Rxi 624 Sil MS 1 μm	N_2_ 2.5	PID	0.1–1.6 (BTEX)
GC-PID [23]	31 × 30 × 20	32	20	∼15	Tube 0.165 cm i.d.	ResSil-B 75 mg	Capillary 15 × 530 (i.d.)	MXT-1 3 μm	A.A. 0.8−2.2	PID	0.002–0.011 (BTEX)
GC-MOS [25]	n. d.	n. d.	5	4	Cavity with micro-pillars 10 × 400 × 5	Zeolite DaY ~ 13 µm	Circular spiral 5 × 100 × 100	PDMS 100 nm	7	MOS	24 (toluene) 5 (o-xylene)
GC-PID [26]	n. d.	n. d.	1	5 (5 comp.)	4 Parallel channels n.d × 400 × 0.6	SWNTs 0.15 mg	Serpentine with micropillars 4 × 350 × 320	OV-101 0.2 µm	A.A. 5	PID	<1 (benzene)
GC-MOS [27]	n. d.	n. d.	n. d.	60 (3 comp.)	n. p.	n. p.	Serpentine 1.6 × 1200 × 600	Porapak Q	A.A.	MOS	5 (benzene)
PEMM-1 [28]	19 × 30 × 14	3.5	1	4 (17 comp.)	2 Cavities (V ~ 9.4 μL) 380 (*d*)	C-B 2.0 mg C-X 2.3 mg	Square spiral 3.1 × 240 × 150	PDMS 0.20 μm	He 3	5 μCR	420–890 (BTEX)
Frog 4000 [29]	25 × 19 × 37	<2.2	0.5	5	n. d.	Silica gel aerogel	4.8 *	PDMS 0.8 μm	A.A.	PID	~ppb
GC-PID [30]	n. d.	n. d.	50	13	n. d.	EtQxBox 10 mg	n. p.	n. p.	A.A. 30	PID	1.25 (benzene)
GC-PID [31]	60 × 50 × 10	<5	2	14.2 (50 comp.)	Cavity 8.15 × 250 × 2.9	C-B 1.135 mg	1D: 10 × 250 (i.d.) 2D: 3 × 250 (i.d.)	1D: Rtx-5MS 2D: Rtx-200 0.25 µm	He 2	µPID	n. d.
GC-CR [32]	20 × 15 × 9	2.1	2.5 min (9 comp.)		2 Cavities (V ~ 9.4 μL)	C-B 2.0 mg C-X 2.3 mg	6 *	PDMS 0.2 µm	n. d.	µCR	n. d.
GC-CMOS [33]	16 × 11 × 11	n. d.	n. d.	n. d.	Cavity with micro-pillars 10 × 250 × 2	Carbon film	Square spiral 3 × 250 × 100	DB-1	n. d.	CMOS	15 (1,3,5-TMB)
iGC3.2 [34]	8 × 10	n. d.	120	10	U shape n.d. × 300 × 1350	C-B + C-X	2 Serpentines 0.30 × 230 (i.d.)	OV-1 0.2 μm	A.A. 0.2	2 CD	10–2 (BTEX)
Zebra GC [35]	15 × 30 × 10	∼1.8	10	<2	Cavity with micro-pillars 13 × 240 × 13	Tenax TA ∼ 200 nm	Serpentine 2 × 70 × 240	OV-1 ∼250 nm	He 1	TCD	∼25 (TEX)
GC-PID [24]	32 × 29 × 14	∼4	1	10	n. p.	n. p.	Capillary 20 × 180 (i.d.)	Rxi 624 Sil MS 1 μm	N_2_ 2.5	PID	0.8–3.2 (BTEX)
GC-MOX [22]	n. d.	n. d.	55	∼12	Ten parallel channels 800 µm depth	QxCav	Square spiral 0.5 × 900 × 900	Carbograph 2 0.2% Carbowax	A.A. 15	MOS	0.1 (benzene)

i.d.: internal diameter, comp.: compounds. n. d.: not defined, n. p.: not present, * only column length is reported. A.A: ambient air. MOS: metal oxide semiconductor. CR: chemiresistor detector. CD: capacitive detector. CMOS: complementary metal oxide sensor. MOX: metal oxide sensor. QxCav: quinoxaline bridged cavitand.

**Table 2:** Calibration equations and limits of detection (LOD) obtained for BTEX with and without the preconcentration module (this work, Nasreddine et al. [24]).

Compound	Calibration Equation	R^2^	LOD 1 (ppb) * (This Work)	LOD 2 (ppb) Nasreddine et al. [24]	Ratio LOD 2/LOD 1
Benzene	y = 2828.2 x	0.9913	0.20	0.72	3.6
Toluene	y = 1206.3 x	0.9777	0.26	1.16	4.46
Ethylbenzene	y = 454.2 x	0.9895	0.49	2.10	4.40
m/p-Xylenes	y = 311.9 x	0.9959	0.80	1.40	1.75
o-Xylene	y = 427.0 x	0.9949	1.70	2.63	1.55

* LOD (ppb) = (3 × lowest injected concentration)/(S/N of the lowest injected concentration).
